# Erectile Dysfunction: Treatments, Advances and New Therapeutic Strategies

**DOI:** 10.3390/brainsci13050802

**Published:** 2023-05-15

**Authors:** Antonio Argiolas, Francesco Mario Argiolas, Giacomo Argiolas, Maria Rosaria Melis

**Affiliations:** 1 Department of Biomedical Sciences, Section of Neuroscience and Clinical Pharmacology, University of Cagliari, 09042 Monserrato, Italy; f.argiolas52@sstudenti.unica.it (F.M.A.); mrmelis@unica.it (M.R.M.); 2 General Medicine Unit, Hospital San Michele, ARNAS“G. Brotzu”, Piazzale Ricchi 1, 09100 Cagliari, Italy; argiolasg@gmail.com

**Keywords:** erectile dysfunction, pharmacological treatments, regenerative strategies, surgical strategies, vacuum erection device, penile prosthesis

## Abstract

Erectile dysfunction (ED) is the inability to get and maintain an adequate penile erection for satisfactory sexual intercourse. Due to its negative impacts on men’s life quality and increase during aging (40% of men between 40 and 70 years), ED has always attracted researchers of different disciplines, from urology, andrology and neuropharmacology to regenerative medicine, and vascular and prosthesis implant surgery. Locally and/or centrally acting drugs are used to treat ED, e.g., phosphodiesterase 5 inhibitors (first in the list) given orally, and phentolamine, prostaglandin E1 and papaverine injected intracavernously. Preclinical data also show that dopamine D_4_ receptor agonists, oxytocin and α-MSH analogues may have a role in ED treatment. However, since pro-erectile drugs are given on demand and are not always efficacious, new strategies are being tested for long lasting cures of ED. These include regenerative therapies, e.g., stem cells, plasma-enriched platelets and extracorporeal shock wave treatments to cure damaged erectile tissues. Although fascinating, these therapies are laborious, expensive and not easily reproducible. This leaves old vacuum erection devices and penile prostheses as the only way to get an artificial erection and sexual intercourse with intractable ED, with penile prosthesis used only by accurately selected patients.

## 1. Introduction

Erectile dysfunction (ED) is defined as the inability (occasional or habitual) to get and maintain an adequate penile erection for a satisfactory sexual intercourse. ED increases with aging, being a dysfunction present in 40% of men between 40 and 70 years, thus affecting millions of men around the world [1]. Among the most frequent causes of ED are neurogenic and vascular factors, which become evident and tend to increase with age, and often in concomitance to other pathologies, such as hypertension, diabetes, atherosclerosis, hyperdyslipidemia and metabolic syndrome. While vascular factors contribute to ED mainly at the local level, neurogenic factors may contribute to ED at all levels of the nervous system, from local supply by the nervous autonomous system to the genital apparatus and to the spinal, supra-spinal and even higher brain centers [2,3]. Both neurogenic and vascular factors interfere with the mechanisms that lead to the relaxation of cavernous smooth muscles, the key event in penile erection. Indeed, relaxation of cavernous smooth muscles allows blood to flow inside the cavernous corpora through helicine arterioles and to compress penile veins against the scarcely dilatable tunica albuginea. This reduces blood outflow from the cavernous corpora and causes penile rigidity, which is increased by voluntary or reflex contractions of the striated bulbospongious and ischiocavernous muscles located at the base of the penis allowing for intromission and sexual intercourse [2,3,4,5,6,7,8].

The aim of this work is to present a brief narrative review of the main progress done in the last 15 years by researchers of different disciplines, mainly at the experimental level, which may contribute to further advancements in the therapy of ED, as this dysfunction has an important impact on the quality of life and well-being of men and partners, whether it occurs in the youth or in the elderly. In fact, although ED therapy has been greatly improved by the discovery of orally active phosphodiesterase type 5 (PDe5) inhibitors in 1992, there are still numerous cases of ED that are unresponsive to these drugs and that require the discovery of new therapeutic strategies to overcome these forms of ED other than the activation of cavernous nitric oxide (NO)–guanylate cyclase (GC)–cyclic guanosine monophosphate (cGMP) pathway by PDe5 inhibition. A brief section on the neurophysiology of erectile function at the central and local levels is also provided before starting with the review of the recent advancements in the field in order to refresh the readers on the physiological and pharmacological basis of the therapy of ED.

## 2. Neural Control of Penile Erection

**Figure 1:**
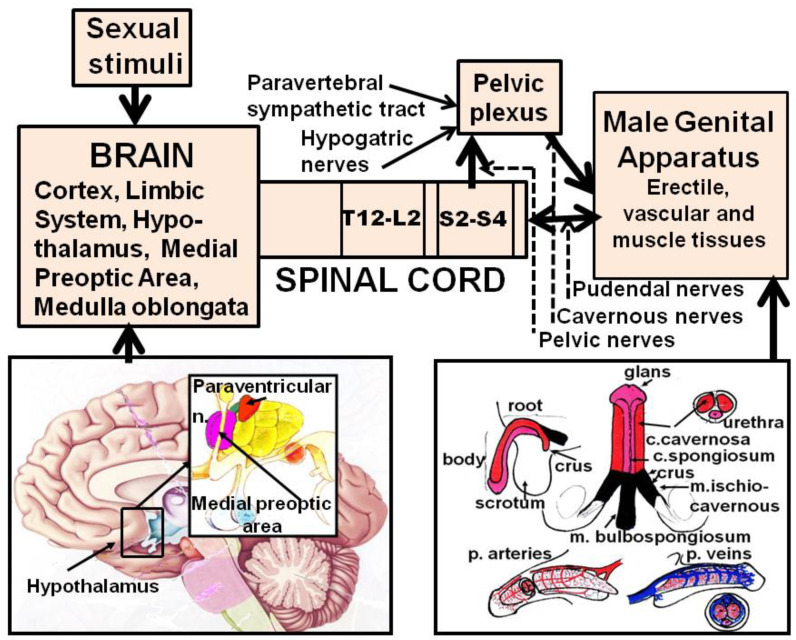
Schematic representation of the neural control of the male genital apparatus. When sexual (visual, auditory, olfactory, tactile, and even imaginative in men) stimuli reach the central nervous system, they activate neural pathways, which are to date still unknown, mediating penile erection and sexual activity. These travel from brain, mainly from the medial preoptic area, hypothalamus and its nuclei (paraventricular nucleus), through the medulla oblongata and the spinal cord, to the genital apparatus. This is innervated mainly by pudendal nerves, which originate from the sacral tract of the spinal cord (S2-S4) and contain the primary afferent sensory and motor pathways to the penis, and by cavernous nerves, which contain the primary efferent sympathetic and parasympathetic pathways originating in the pelvic plexuses. These receive neural inputs by hypogastric nerves, originating in the thoracic-lumbar tract of the spinal cord (T12-L2), and by pelvic nerves originating in the sacral tract of the spinal cord (S2-S4). Pelvic plexuses also receive post-gangliar fibers, which originate from the paravertebral sympathetic ganglia of the thoracic-lumbar tract of the spinal cord (T11-L2). For details, see references [1,2,3,4,5,6,7,8,9,10,11,12,13,14,15,16,17,18,19,20,21,22,23,24,25,26,27,28,29,30].

When sexual stimuli reach the central nervous system, neural pathways are activated that convey sexual information from the higher brain centers through the spinal cord and the autonomous nervous system to the genital apparatus to induce penile erection ([2,7,8] and references therein) (Figure 1).

**Figure 2:**
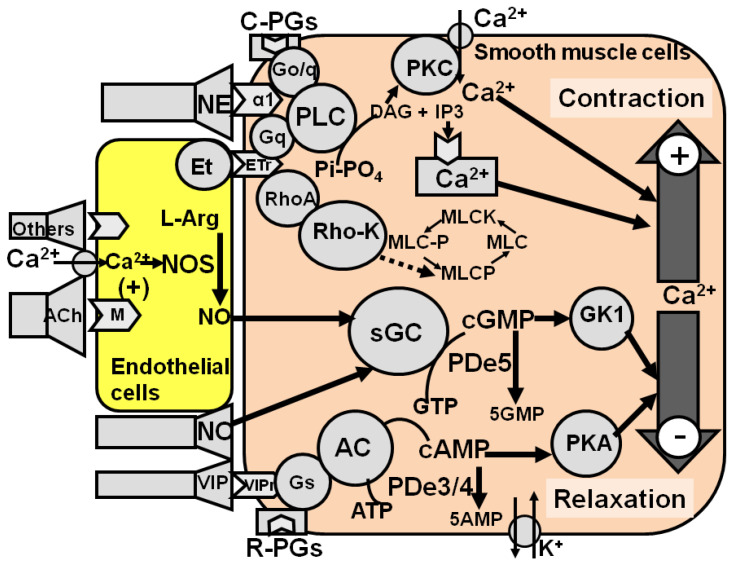
Schematic representation of local mechanisms controlling the tone of cavernous smooth muscles. When the penis is flaccid, cavernous smooth muscles are contracted by noradrenaline (NE), which acts on α_1_ receptors to increase intracellular free Ca^2+^ by activating the phospholipase C/inositol triphosphate (IP_3_)/diacylglycerol (DAG) pathway, by endothelins (ET) and contracting prostaglandins (C-Pgs), which act with mechanisms similar to that of NE, and by the RhoA-Rho kinase system, which keeps myosin light chain (MLC) phosphorylated (MLC-P) by inhibiting MLC phosphatase (MLCP), facilitating the myosin/actin interaction. Relaxation occurs when sexual stimuli increase nitric oxide (NO) levels in cavernous smooth muscles. NO is released from nitrergic nerve endings and from endothelial cells containing endothelial NO synthase (activated to produce NO by the stimulation of muscarinic (M) receptors by Ach), activating soluble guanylate cyclase (sGC) and increases cyclic guanosine monophosphate (cGMP). cGMP acts on protein kinase GK1 to decrease intracellular free Ca^2+^, facilitating relaxation and penile erection. cGMP action is terminated by phosphodiesterase type 5 (PDe5). Relaxation of cavernous smooth muscles is also obtained with peptides such VIP and relaxing prostaglandins (R-Pgs) that activate adenylate cyclase (AC) and increase cyclic adenosine monophosphate (cAMP). cAMP acts on protein kinase A (PKA), decreases intracellular free Ca^2+^, and facilitates relaxation and penile erection. cAMP action is terminated by PD type 3/4 (PDe3/4). Drugs that potentiate relaxation or reduce contraction mechanisms are suitable candidates for ED treatment.

Within the cavernous corpora, three main neural controls have been characterized in detail: the first is noradrenergic stimulatory, which maintains cavernous smooth muscles contracted, thus leading to the penis being flaccid (normal condition); the second is cholinergic inhibitory, which causes relaxation of cavernous smooth muscles and facilitates erection; and the third is nonadrenergic–noncholinergic inhibitory, which facilitates relaxation while erection and is mediated by NO [9,10], although the role for other endogenous compounds cannot be ruled out [7,8] (Figure 2).

Briefly, under normal conditions, cavernous smooth muscles are contracted (e.g., the penis is flaccid). The main stimulatory control is mediated by noradrenaline, which acts on G_o/q_ protein α1 receptors located in cavernous smooth muscle cells coupled to phospholipase C, which produces inositol triphosphate and diacylglycerol from phosphatidyl-inositol. Inositol triphosphate in turn releases Ca^2+^ ions from intracellular stores, while diacylglycerol activates protein kinase C (PKC) leading to an increase in intracellular free Ca^2+^ ions, causing contraction. Contributions to keep cavernous smooth muscles contracted are also provided by peptides (i.e., endothelins) and by contracting prostaglandins, which act with mechanisms similar to that of noradrenaline, and by the RhoA-Rho kinase system, which, when activated, keeps the myosin light chain phosphorylated by inhibiting myosin light chain phosphatase, thus facilitating the interaction between myosin and actin (see [8]). Relaxation of cavernous smooth muscles takes place when sexual stimuli activate the inhibitory tone mediated by acetylcholine and by NO to overcome contraction, leading to penile erection. Acetylcholine acts on muscarinic receptors located in endothelial cells, whose stimulation lead to increase the concentration of intracellular free Ca^2+^ ions. This in turn activates NO synthase, to produce NO. Endothelium-derived NO diffuses to smooth muscle cells and, together with NO released from nerve endings, activates soluble guanylate cyclase (GC). Soluble GC increases cGMP that acts on protein kinase GK1, causing a decrease in intracellular Ca^2+^ ions and inducing relaxation. cGMP action is terminated by PDe5. Cavernous smooth muscles also contain G_s_-coupled protein receptors for endogenous peptides (i.e., vasoactive intestinal peptide, calcitonin gene-related peptide) and relaxing prostaglandins coupled to adenylate cyclase (AC). The activation of these receptors increases the concentration of cyclic adenosine monophosphate (cAMP), which acts on protein kinase A, decreases intracellular free Ca^2+^ ions and facilitates relaxation. cAMP action is terminated by PDe3/4 (Figure 2). Alterations of these local mechanisms at various levels (from the synthesis and release of neurotransmitters, peptides, autacoids and others to their interaction with specific receptors, enzymes and subsequent transduction mechanisms activated by second messengers cGMP and cAMP, Ca^2+^ ions and others) may be targeted to cause relaxation of cavernous smooth muscles and facilitation of penile erection (see [2,4,5,6,8,11,12]) (Figure 2).

In contrast to the detailed knowledge of local mechanisms controlling the tone of cavernous smooth muscles, at the central level, very little is known of the neural pathways conveying sexual information from the brain to the genital apparatus to induce penile erection even if a few brain areas involved in erectile function have been identified. These include the medial preoptic area (MPOA), the hypothalamus and its nuclei ((i.e., the paraventricular nucleus (PVN)), the ventral tegmental area (VTA), the nucleus accumbens, the hippocampal formation, the amygdala, the bed nucleus of the stria terminalis, the nucleus paragigantocellularis of the reticular formation and the spinal cord [2,5,6,13,14,15,16,17,18,19,20,21,22,23]. In these areas, different neurotransmitters and neuropeptides that can influence erectile function have been identified. Among these are dopamine, oxytocin, NO, excitatory amino acids, adrenocorticotropin (ACTH) and α-melanocyte-stimulating hormone (α-MSH)-related peptides, growth hormone (GH)-releasing peptides and VGF-derived peptides that facilitate erection, while GABA, opioid peptides, endocannabinoids and serotonin (although not always) inhibit this sexual response [6,7,20,21,22,23,24,25,26,27,28,29,30]. Thus, from a theoretical point of view, neurotransmission of all the above neurotransmitters/neuropeptides might be targeted for influencing penile erection. However, in spite of numerous preclinical studies supporting this possibility, so far only scarce clinical evidence has proved the feasibility of central strategies for ED therapy. Indeed, only apomorphine, a centrally acting mixed dopamine D1/D2 receptor agonist and yohimbine have been introduced into clinical practice for the treatment of ED, with very modest success when compared to that of orally active and locally acting phosphodiesterase type V inhibitors (see Section 3.1.2.2).

## 3. ED Treatments

ED treatments are aimed to restore and maintain an adequate penile erection for sexual intercourse. The most common are pharmacological treatments, which are based on the use of pro-erectile drugs, administered systemically or locally. When pro-erectile drugs are found inefficacious, other strategies must be used to treat ED. This often requires the identification of the main cause of the dysfunction and leads to the search of “restorative” and/or “regenerative” strategies of erectile function. Among these new strategies are not only stem-cell- and plasma-enriched-platelets-based therapies aimed at the restoration of cavernous endothelial and smooth muscle cells and nerves; gene therapy, aimed at potentiating the activity of key genes involved in the relaxation of cavernous smooth muscles (both strategies have been initially developed in the field of regenerative medicine); and low-intensity extracorporeal shock wave therapies, which also are thought to work by activating regenerative cellular processes at the cavernous tissue level, but also invasive surgical procedures at the level of the pelvic vascular (arterial and/or venous) bed aimed at the restoration of a satisfactory in- and out-blood flow during penile erection and intracavernous applications of botulinum neurotoxin A. The use of old vacuum erection devices and surgical implants and of technologically advanced penile prostheses, which can simulate the natural erection process, are still available for intractable ED.

### 3.1. Pharmacological Strategies

Pharmacological strategies for ED treatment may be divided into two categories: those acting at the local level, and those acting at the central level. At the local level, they may be of two kinds: those aimed at facilitating relaxation and those aimed at reducing the contraction of cavernous smooth muscles. At the central level, two kinds of strategies for ED are also possible: The first includes those aimed at increasing the activity of neurotransmitters and/or neuropeptides that facilitate penile erection, and the second include those aimed at reducing the activity of neurotransmitters and/or neuropeptides that inhibit this sexual response.

#### 3.1.1. Local Strategies

As recalled above, two local strategies are available: one aimed at facilitating relaxation and the other at reducing the contraction of cavernous smooth muscles by acting on mechanisms involved in these processes (see Section 2) ([8,11,12] and references therein) (Table 1).

The relaxation of cavernous smooth muscles takes place when the stimulatory sympathetic adrenergic tone is overcome by the inhibitory nitrergic and parasympathetic cholinergic tones originating from the cavernous corpora from the sacral spinal cord. As acetylcholine acts by increasing NO production from endothelial cells laying over cavernous smooth muscle cells, this makes NO the main relaxing compound of cavernous smooth muscles. As NO induces its relaxing effect by activating soluble GC, thereby increasing the concentration of cGMP, the main controller of intracellular free Ca^2+^ ions levels, which determines the final relaxed or contracted status of cavernous smooth muscles, it is universally accepted that the easiest way to facilitate the relaxation of cavernous smooth muscles is the increasing of the NO–GC–cGMP signaling pathway activity, at least when neural pathways and endothelial cells laying over cavernous smooth muscle cells are still operative. In fact, every drug able to activate this pathway in the cavernous corpora, would elicit relaxation of cavernous smooth muscles and penile erection, while drugs that inhibit this pathway would reduce such responses. Accordingly, the most successful pharmacological treatment of ED today available relies on the increase of the activity of cavernous NO–GC–cGMP signaling pathway obtained by increasing cGMP levels after the inhibition of PDe V with rather selective and potent orally active inhibitors of this enzyme [31]. This is a consequence of the selective localization of the PDe5 enzyme isoform in cavernous tissue and of the scarce effect of these drugs on other known PDe enzyme isoforms found in numerous tissues and organs, including cavernous smooth muscles [31]. In fact, such selectivity combined with usually modest collateral effects cannot be obtained with other drugs that increase the activity of the NO–GC–cGMP signaling pathway (e.g., NO donors, soluble GC stimulators/activators and stable cGMP analogues), unless these drugs are given intracavernously.

Alternatively, the relaxation of cavernous smooth muscles may be obtained by stimulating neurotransmitter/neuropeptide/autacoid receptors present in the cavernous corpora coupled to G_s_ proteins for AC activation, thus increasing cAMP levels in the cavernous corpora smooth muscles with vasoactive intestinal peptide (VIP), calcitonin-related peptide and mainly with the relaxing prostaglandin E1 [7]. cAMP facilitates the relaxation of cavernous smooth muscles through the activation of protein kinase A, which causes a decrease in intracellular free Ca^2+^. cAMP action is terminated by a PDe3/4. Although experiments with knockout mice on the cGMP-dependent protein kinase GK1 have shown that cAMP cannot replace cGMP in the physiological relaxation of cavernous smooth muscles [32], the activation of this pathway, either by activating receptors coupled to this system or by inhibiting PDe3/4, is an efficacious pharmacological strategy for ED treatment. However, since the AC–cAMP signaling pathway is ubiquitous in all tissues and organs, drugs acting on this pathway must be given intracavernously, as in the case of prostaglandin E1 and its analogue alprostadil, which are administered efficaciously and with scarce/minor side effects, and the opium alkaloid papaverine, which increases cAMP levels by inhibiting PDe3/4 [7].

Relaxation of cavernous smooth muscle can also be obtained with drugs that block α_1_-adrenergic receptors and endothelin receptors, and drugs that inhibit the RhoA/Rho kinase system. In fact, these drugs induce relaxation of cavernous smooth muscles in vitro and often facilitate erection in vivo [7,11,12]. This strategy is limited by the fact that similar mechanisms take place in all smooth muscles of the body. Thus, achievement of selective effects on cavernous tissue is impeded after systemic administration of these drugs, although numerous compounds that interact with these systems are available. To mention but a few, α_1_-adrenergic receptor antagonists given systemically are able to induce erection and also priapism, but significantly decrease systemic blood pressure, inducing side effects incompatible with sexual activity [7]. This also occurs with RhoA/Rho-kinase inhibitors, which inhibit the contraction of cavernous smooth muscles induced by noradrenaline in vitro and induce penile erection in rodents in vivo [33], but significantly reduce systemic blood pressure [7,11,12]. Thus, the above compounds must also be injected intracavernously in order to obtain a selective effect on penile erection. This strategy is used for the intracavernous treatment of ED, based on the local injection of drugs, mainly α_1_-adrenergic antagonists, usually given in combination with other drugs that cause relaxation of cavernous smooth muscles with different mechanisms, e.g., prostaglandin E1, alprostadil and papaverine, and recently also botulinum neurotoxin A (see Section 4.3).

##### 3.1.1.1. Local Treatments

As outlined above, the main local targets for the pharmacological therapy of ED are: (1) noradrenergic α_1_ receptors and endothelin receptors, whose stimulation keeps the cavernous muscles contracted by influencing either the amount of free Ca^2+^ through the phospholipase C/diacylglicerol/phosphatidilinositol signaling pathway and the activity of the RhoA/Rho-kinase system; (2) the different steps of the NO–GC–cGMP signaling pathway, from the synthesis of NO by the Ca^2+^-calmodulin-dependent NO synthase to GC to the inactivation of cGMP by PDe5; and (3) receptors of neuropeptides, autacoids and others endogenous compounds linked to the AC–cAMP signaling pathway and the inactivation of cAMP by PDe3,4. Both cGMP and cAMP regulate Ca^2+^ levels in cavernous smooth muscles, determining its contracted or relaxed status (Figure 2). The most studied target is the NO–GC–cGMP signaling pathway and, in particular, the inactivation of cGMP by PDe5. All other local targets, i.e., α_1_ receptors, endothelin receptors, the RhoA/Rho-kinase system, NO synthase, GC and receptors that activate the AC–cAMP signaling pathway, are suitable for the intracavernous therapy of ED (see above). Botulinum neurotoxin A has been recently added to the list of drugs that facilitate cavernous smooth muscle relaxation when injected in the cavernous tissues, however, botulinum neurotoxin A will be discussed with the non-surgical therapies of ED, as this treatment cannot be considered as a self-treatment as it must be performed in appropriate medical settings (see Section 4.3).

##### 3.1.1.2. PDe5

The PDe5 enzyme isoform is selectively localized in the cavernous smooth muscles. Together with the absence of important collateral effects, this has made orally active PDe5 inhibitors the most efficacious drugs commercially available for ED therapy [31,43,44]. Among these are globally available sildenafil, vardenafil, tadalifil and avenafil [45], while mirodenafil [46], udenafil [47] and lodenafil [48] are available (the first two only in Korea, and the latter only in Brazil). The globally available drugs differ, chemical structure apart, only in their pharmacokinetic properties [31,43]. Indeed these drugs bind to the catalytic subunit of the enzyme inhibiting the inactivation of cGMP, thus allowing greater activation of protein kinase G (cGKI) and greater relaxation of cavernous smooth muscle. One of the limits of these drugs is represented by physiopathological conditions, which lead to low levels of NO at the penile level. This occurs in several organic causes of ED, such as during diabetes, where endothelial function is markedly compromised and/or during hypercholesterolemia [49,50,51,52]. To overcome this impasse, classic NO donors and even L-arginine, the natural substrate of NO synthase, have been used alone and in combination with PDe5 inhibitors for the treatment of ED, but with scarce and often contrasting results (reviewed in [22]). This led to the synthesis of NO-releasing PDe5 inhibitors, and one of these compounds, NCX 911, a NO-releasing sildenafil derivative, has been reported to be more effective than the base compound in relaxing human and rabbit cavernous smooth muscles [53,54,55,56]; however, no attempt to study the effect of this compound in vivo has been reported. This is important because NO-releasing compounds are well known for their potency in inducing vasodilatation and decreasing blood pressure, which may be incompatible with the ED therapy. This also applies to light-controllable NO donors, which release NO when exposed to a given wavelength of light and have been found to be able to induce smooth cavernous smooth muscle relaxation in vitro and penile erection in vivo in anesthetized rats [57,58,59,60]. Although light-controllable NO donors may be efficacious for non-invasive ED therapy, it has still to be found how to maintain penile erection once intromission has occurred and light cannot activate NO release anymore and how to synthesize light-controllable NO donors that are less citotoxic than those actually available, due to the formation of highly reactive peroxynitrite ions [59,60]. The latter effect may also occur with NO donors loaded on nanoparticles and nanoemulsions, which can be used to potentiate a better adsorption of NO donors across the penile gland and/or skin [61]. Further progress in this research field is required for these type of drugs.

##### 3.1.1.3. GC

NO is the main activator of GC. However, the GC molecule also contains a NO-independent site [62,63,64], which may be activated by compounds such as YC-1 [3-(5′-hydroxymethyl-2′-furyl)-1-benzyl Indazole] [65] and by BAY 41-2272 [5-cyclopropyl-2-[1-(2-fluorobenzyl)-1H-pyrazolo[3,4-b]pyridin-3-yl]pyrimidin-4-ylamine] and BAY 60-2770 (4-[((4-Carboxy-butyl)-{2-[5-fluoro-2-(4′-trifluoromethyl-biphenyl-4-ylmethoxy)-phenyl]-ethyl}-amino)-methyl]-benzoic acid) [66,67,68,69] (for a review on NO-independent stimulators and activators of soluble GC in lower urinary tract symptoms and ED, see [36]). These drugs are able to relax human and rabbit cavernous and vascular smooth muscles, effects which are mediated by increased cGMP levels [70,71,72]. However, when given systemically, these drugs also markedly reduce blood pressure and inhibit platelet aggregation [73,74], (for a review on soluble GC activators and blood pressure, see [75]).

##### 3.1.1.4. Arginase

NO levels are influenced by the activity not only of NO synthases but also of arginases. Two arginases are known: arginase 1, a cytosolic enzyme present mainly in the liver, where it plays a key role in eliminating nitrogen formed during amino acid and nucleotide metabolism via the urea cycle, but also in vascular tissues, and arginase 2, a mitochondrial enzyme present in several tissues and in the vasculature [76]. These two homologue enzymes convert L-arginine to urea and L-ornithine, and compete with NO synthases for L-arginine. Thus, increased arginase activity may reduce NO production in the vascular endothelium in normal and disease states [37], including ED. Two recent studies have found that arginase 2 levels are higher in clinical ED [77,78] and that genetic variations in arginase 1 differentially affect the severity of clinical ED [77]. Together with the presence of arginases in the human penis [79], this makes arginases a realistic target for the therapy of vascular ED, as it occurs in diabetes and atherosclerosis [76,80,81]. Several compounds have been identified that inhibit arginase activity, such as boron-based amino acid derivatives; α-difluoromethylornithine (DMFO); and Nω-hydroxy-nor-L-arginine (nor-NOHA) [82] or compound isolated by plants, such as sauchinone [83]; salvianolic acid B [84]; piceatannol-3-O-β-D-glucopyranoside (PG) [85]; and obacunone [86] (for a review on Arginase inhibitors, see [38,87]). Only some of these compounds have been studied in in vitro settings and in vivo in a few animal species, revealing complex physicochemical and pharmacokinetic profiles that make their use in humans challenging. This justifies, in part, the absence of arginases inhibitors in clinical practice and reveals that more studies, at least with the most active compounds, are required to determine how they inhibit the activity of the two arginases in order to identify the doses and routes of administration to be tested in vascular pathologies, including ED, as has been done with other polyphenols (resveratrol and quercetin) in cardiovascular diabetology [88,89,90].

##### 3.1.1.5. RhoA-Rho Kinase System

The RhoA/Rho kinase system also plays an important role in the maintenance of penile flaccidity, as inhibition of its tone causes the relaxation of cavernous smooth muscles, thus initiating penile erection [91,92]. Several drugs able to inhibit this system are available, with Y-27632 (trans-4-[(1*R*)-1-Aminoethyl]-*N*-4-pyridinylcyclohexanecarboxamide dihydrochloride) and HA-1077 (fasudil, hexahydro-1-(5-isoquinolinylsulfonyl)- 1H-1,4-diazepine, dihydrochloride) being two of the most studied ones. These two drugs induce relaxation of cavernous corpora tissues in vitro and induce penile erection in vivo in male rats [33,34,93,94,95,96,97]. Fasudil has been approved for cerebral vasospasm therapy, and one of its derivative has been approved for the treatment of glaucoma in Japan [98]. SAR407899 (6-(4-piperidinyloxy)-1(2H)-isoquinolinone) and azaindole-1 (6-chloro-N4-{3,5-difluoro-4-[(3-methyl-1H-pyrrolo[2,3-b]pyridin-4-yl)oxy]-phenyl}pyrimidine-2,4-diamine) are two other RhoA/Rho kinase inhibitors able to induce penile erection in diabetic rabbits and rats (the former in diabetic rats, and the latter in a NO-independent manner) [99,100]. Despite numerous studies supporting the role of the RhoA/Rho kinase system in improving erectile function in pathological conditions, such as diabetes, hypertension and hypercholesterolemia [91], and the improving effects of alternative herbal medicine, likely mediated, at least in part, by compounds that inhibit the RhoA/Rho kinase system [101], no drug capable of inhibiting this system is currently under investigation for ED therapy. The main problem with the known inhibitors of the RhoA-Rho kinase system is that this system is present in all vascular beds, and all these drugs given systemically have also potent vasorelaxing properties, which lead to a marked decrease in systemic blood pressure [102,103].

##### 3.1.1.6. α1-Adrenergic Receptors

As recalled above, noradrenaline is the main stimulatory neurotransmitter which keeps cavernous smooth muscles contracted and the penis flaccid by acting on α_1_-adrenergic receptors coupled to the phospholipase C/diacylglycerol/phosphoinositol signaling pathway. This promotes Ca^2+^ mobilization from intracellular stores and activation of myosin light chain kinase, which phosphorylates myosin light chains and initiates the contractile response of cavernous smooth muscle in conjunction with actin. Accordingly, the blockade of α_1_-adrenergic receptors with the α_1_ receptor antagonist phentolamine induces relaxation of the cavernous smooth muscles in vitro and penile erection when injected intracavernously in men [104]. Similar results are obtained with other α_1_-adrenergic receptor antagonists, such as phenoxybenzamine and moxisylite [42], but phentolamine is the only α_1_-adrenergic receptor antagonist used in the intracavernous therapy of ED, usually in combination with other drugs (i.e., prostaglandin E1, alprostadil and/or papaverine) [105] (see below). However, even when given intracavernously, this drug often produces orthostatic hypotension, tachycardia and, although rarely, also priapism [106,107]. These effects (except priapism) are usually seen with α_1_-adrenergic receptor antagonists given systemically and have been used for a long time as a monotherapy for the control of idiopathic hypertension, benign prostatic hyperplasia and associated bladder neck obstruction, voiding and storage, which also induce alterations in sexual, mainly ejaculatory function [108].

##### 3.1.1.7. Prostanoid Receptors

Prostaglandins exert numerous effects on cavernous smooth muscles, depending on the type of prostaglandin. One of these, prostaglandin E1 induces cavernous smooth muscle relaxation and penile erection when injected intracavernously. Prostaglandin E1 activates the AC/cAMP pathway, thus increasing cAMP levels, which activates protein kinase A, facilitating Ca^2+^ mobilization from intracellular stores and the relaxation of cavernous smooth muscles [7]. This makes this prostaglandin, given alone or in combination with other drugs (see Section 3.1.1.6 and Section 3.1.1.8), one of the most used drugs for intracavernous ED therapy when PDe5 treatment is found to be inefficacious [109]. Alprostadil is a synthetic prostaglandin E1 derivative that can be also used topically, intraurethrally or intracavernosally and apparently has an established efficacy and a high safety profile without important systemic adverse events [105,106,110,111].

##### 3.1.1.8. PDe3/4

Like prostaglandins, endogenous peptides in the cavernous tissue, such as vasoactive intestinal peptide (VIP) or calcitonin-gene related peptide (CGRP), injected intracavernously induce penile erection by activating receptors the AC/cAMP pathway. The facilitatory effect of cAMP on the relaxation of cavernous smooth muscles is terminated by its inactivation by PDe3/4. The blockade of these enzymes by drugs injected intracavernously, such as papaverine, induces penile erection. The intracavernous injection of a combination of prostaglandin E1, phentolamine and papaverine is the most common therapy for the local treatment of ED and is usually used when oral PDe5 inhibitors are found to be inefficacious [105,107,108,109,110,111,112].

#### 3.1.2. Central Strategies

The facilitation of penile erection by drugs acting at the central level is a possible and fascinating, although it is a very complicated strategy for the ED treatment. As recalled above, several neurotransmitters and neuropeptides that facilitate penile erection have been identified in different brain areas [2,6,13,14,15,16,17,25,27]. Although drugs that stimulate the activity of these neurotransmitters and/or neuropeptides either by acting on their receptors or with other synaptic mechanisms are available, very few of them induce penile erection when given systemically (Table 2). So far, the only drugs targeting the central nervous system that have been used for the ED treatment in men are apomorphine (see [8]), the α_2_-adrenoceptor antagonists yohimbine and delequamine [113,114,115], the α-melanocyte-stimulating hormone (α-MSH) receptor agonist melanotan II [116] and the serotonin reuptake inhibitor trazodone [117,118]. As for apomorphine, a large amount of preclinical data show that apomorphine facilitates penile erection and sexual activity by acting on dopamine receptors of the D2 family in different brain areas.

As its pro-erectile effect is shared by many other D2 receptor agonists, these dopamine receptors are one of the most important central molecular target for the ED treatment (see below) [23]. Yohimbine and delequamine have also been tested for their effect on ED. These drugs block presynaptic α_2_ adrenoceptors, increase the release of noradrenaline from central nerve endings and induce an increased sexual arousal together with an increased erectile response and an increased volume of ejaculate ([113,114,115] and references therein). Trazodone also has been tested for ED, but its use is complicated by sedative effects that are incompatible with sexual arousal and sexual performance [118]. Melanotan II is a cyclic α-MSH peptide derivative that underwent clinical investigation for ED treatment. The compound induces its pro-erectile effect apparently by acting on central hypothalamic melanocortin (MC) receptors not only in laboratory animals but also in men after systemic administration ([116] and references therein).

##### 3.1.2.1. Central Targets

**Figure 3:**
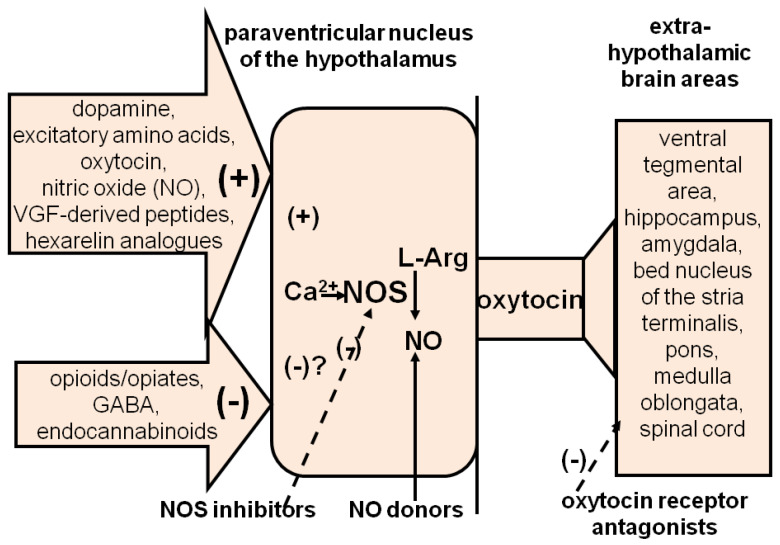
Oxytocinergic neurons are a target for the central therapy of ED. These neurons, originating in the PVN and projecting to the spinal cord and extra-hypothalamic brain areas, mediate penile erection. When activated by neurotransmitters, such dopamine, excitatory amino acids, NO, oxytocin, hexarelin peptide analogues, VGF peptides or by the blockade of CB1 receptors in the PVN, they facilitate penile erection, while when inhibited, for instance by gamma-aminobutyric acid (GABA), opioid peptides and endocannabinoids, this sexual response is reduced. NOS = NO synthase. References are found in Section 3.1.2. (Section 3.1.2.2 and Section 3.1.2.3).

At variance from the local level, much less is known of the pharmacological targets suitable for the therapy of ED at the central level. This in spite of the fact that drugs that act as neurotransmitter agonists and/or antagonists and a few neuropeptides induce penile erection when given systemically and/or into specific brain areas [6,13,14,15,16,17,20,21,22,23,25,26,27,28,29,30]. Although preclinical studies revealed many likely molecular targets for ED treatment, it is unfeasible to treat ED by injecting drugs directly into brain nuclei controlling penile erection in order to obtain a selective pro-erectile effect. Thus, the only way of treating ED with centrally acting drugs is to use systemic drugs that act in these brain areas, particularly those from which neural pathways conveying sexual stimuli from the brain to the genital apparatus originate. One of these is oxytocinergic, originating in the PVN, which projects to extra-hypothalamic brain areas and to the spinal cord, and facilitates erection when activated [6,15,20,21,22,23,27,28,29,30] (Figure 3).

As several neurotransmitters and neuropeptides facilitate or inhibit penile erection by activating or inhibiting this oxytocinergic pathway at the PVN level, receptors of these neuromodulators may all be considered targets for ED therapy by centrally acting drugs [21,22,23,30]. Among these are dopamine, excitatory amino acid and oxytocin receptors, whose activation facilitates the erectile response; GABA, opioid and cannabinoid receptors, whose block would be expected to increase penile erection by reducing the inhibitory effects of these neuromodulators on PVN oxytocinergic neurons; and the receptors of hexarelin- and VGF-related peptides, which facilitate penile erection when injected into the PVN by increasing central oxytocinergic neurotransmission [20,21,22,29,30] (Figure 3). Dopamine receptor agonists, excitatory amino acid receptor agonists and oxytocin may also facilitate penile erection and sexual behavior by acting in other brain areas [20,21,22,23,30]. ACTH-MSH peptide and non-peptide derivatives also induce penile erection by acting centrally, but with mechanisms unrelated to oxytocinergic neurotransmission [29,30,116]. As the role of many of the above neurotransmitters and neuropeptides and of their receptors in the control of erectile function and sexual behavior has been recently reviewed in detail [21,22,23,30], only the more recent advances on the receptors of dopamine, oxytocin and ACTH-MSH peptides as central targets in the therapy of ED will be considered here.

##### 3.1.2.2. Dopamine Receptors

Dopamine receptors have been considered a central target for the therapy of ED since 1980 when dopamine receptor agonists were found able to facilitate erectile function and male sexual behavior in rodents ([23,25] and references therein). Since then, a large amount of preclinical evidence has shown that dopamine activity is increased in the PVN, MPOA and nucleus accumbens when penile erection occurs in physiological contexts, including copulation [13,119,120,121], and that different drugs that increase dopamine neurotransmission induce penile erection in laboratory animals and in men [23,25,122]. As already recalled, the pro-erectile effect of dopamine agonists is mediated by the stimulation of dopamine receptors of the D2 family located mainly in the PVN, MPOA and nucleus accumbens. As for the PVN, dopamine agonist-induced penile erection is abolished by the prior injection of D2 into the PVN but not D1 receptor antagonists [123]. D2 receptor agonists induce penile erection when injected into the PVN by activating NO synthase in the cell bodies of oxytocinergic neurons. The increase in NO production in turn activates oxytocinergic neurons to release oxytocin in the spinal cord and extra-hypothalamic brain areas, leading to penile erection ([23,27,28,29,30] and references therein) (see Section 3.1.2.2).

The ability of dopamine D2 receptor agonists to induce penile erection in laboratory animals and in men led to the introduction of apomorphine, a mixed dopamine receptor agonist, into clinical practice for the treatment of ED in 2000 [124]. The drug has proved to be scarcely effective in men when compared with orally active PDe5 inhibitors, mainly because of its side effects shared by many other dopamine receptor agonists, e.g., nausea and vomiting mediated by dopamine receptors located in the chemo-trigger zone of the medulla oblongata [8]. In the 1990s, molecular biology studies revealed that several D1 and D2 receptor subtypes belong to the D1 and D2 receptor families. Three D2-like receptors (D_2_, D_3_ and D_4_) have been identified and different variants cloned ([125,126,127] and references therein) (Table 3). This led to the synthesis of molecules that act selectively on these different receptor subtypes, with the aim to characterize their role in mental disorders ([128] and references therein).

One of these molecules, ABT 724 [2-(4-pyridin-2-ylpiperazin-1-ylmethyl)-1H-benzimidazole], a selective dopamine D_4_ agonist, was found to be able to induce penile erection when given systemically and into the lateral ventricles in male rats, with a potency similar to that of apomorphine [129]. More importantly, unlike apomorphine, ABT 724 was unable to induce emesis in the ferret [130]. Other D4 receptor agonists were soon found to be able to induce penile erection when given not only systemically and into the lateral ventricles but also into the PVN by activating oxytocinergic neurons with a mechanism similar to that of apomorphine and other D2-like receptor agonists [131,132,133,134]. Since L 745,870 [3-((4-(4-Chlorophenyl) piperazin-1-yl)-methyl)-1H-pyrrolo-2,3-b-pyridine], a selective D_4_ receptor antagonist, was found to be able to abolish D_4_ agonist-induced penile erection [131,132] and functional D_4_ receptors are present in the cell bodies of oxytocinergic neurons in the PVN [134,135,136], this led to a search for D_4_ receptor agonists that are able to induce penile erection but are devoid of collateral emetic effects making, D_4_ receptor agonists a future yet to be tested as a target for ED treatment. Despite some controversy on the facilitatory role of D_4_ receptors on erectile function [137,138], the lastest available studies confirm that (1) D_2_ receptors in the PVN play the main role in penile erection induced by classical dopamine agonists, such as apomorphine and pramipexole, (2) the selective stimulation of D_4_ receptors in the PVN is able to induce penile erection per se, although with a potency lower than that of apomorphine [139,140], and (3) D_4_ receptor agonists given systemically facilitate sexual behavior in male rats with a profile similar to that of apomorphine and other dopamine agonists [141].

Dopamine facilitates penile erection and sexual activity by acting in the MPOA and nucleus accumbens. As for the MPOA, dopamine agonists injected into the MPOA induce penile erection and facilitate penile reflexes and ejaculation [13], and a facilitative role of NO on the above functions has been also found in this area [142], as was found in the PVN. However, the neural pathways mediating these facilitative dopaminergic effects are still unknown [23], although experimental evidence suggest that dopamine in the MPOA may also contribute to the activation of oxytocinergic neurotransmission, as was found in the PVN [143].

As for the nucleus accumbens, this is another brain area in which dopamine activity is increased during the anticipatory and consummatory phase of sexual activity [120,121]. This nucleus contains the nerve endings of mesolimbic dopaminergic neurons that have their cell bodies in the VTA and play a key role in motivation and reward for natural stimuli (e.g., food and sex). As the increases in dopamine activity during sexual activity take place almost concomitantly across the brain, it is likely that a concerted action of dopaminergic systems takes place in the central nervous system during sexual activity. This suggests the existence of a complex neural circuit involving different neurotransmitters (dopamine, glutamic acid, NO) and neuropeptides (oxytocin), which contributes to the concomitant manifestation of the different aspects of sexual behavior, from sexual motivation to sexual performance [23,144,145,146,147,148]. This circuit also allows an interaction between mesolimbic and incertohypothalamic dopaminergic systems, as confirmed by microdialysis experiments, showing that activation of D_2_ and D_4_ receptor subtypes in the PVN by doses of dopamine agonists that induce penile erection increases the release of dopamine in the nucleus accumbens [144,145]. The activation of mesolimbic dopaminergic neurons that follows the stimulation of dopamine receptors in the PVN is mediated by oxytocinergic neurons that project from the PVN directly to the VTA and/or indirectly to the ventral hippocampus [145,146,147,148]. In the VTA, oxytocinergic neurons make direct contact with the cell bodies of mesolimbic dopaminergic neurons [144,145], while in the ventral hippocampus, oxytocinergic neurons act on excitatory glutamatergic neurons that project to the VTA and activate mesolimbic dopaminergic neurons [146,147,148,149] (see also Section 3.1.2.3). Together the findings reviewed above confirm that D_4_ receptors may be considered a valuable pharmacological target to treat ED in men and open the way for ED treatment with D_4_ receptor agonists devoid of emetic properties.

##### 3.1.2.3. Oxytocin Receptors

The facilitatory role of oxytocin on erectile function and sexual behavior was discovered in 1985, when t peptide was found to be able to facilitate male sexual behavior in rodents when injected systemically [150] and induce penile erection when injected into the lateral ventricles or into the PVN of male rats at doses as low as 3 ng (e.g., 3 picomole) [151,152,153]. Since then, a large amount of preclinical studies supporting the main role of oxytocin in erectile function and sexual behavior has appeared and has continued to appear. As this literature has been reviewed recently [21], only a few points supporting that oxytocin receptors may be considered a central target for the therapy of ED will be considered here. As recalled above, oxytocin induces penile erection when injected into the PVN of male rats. Here, the peptide acts as a potent activator of its own neurons, mediating penile erection [6,27,28,29,30] (Figure 3). The oxytocin effect is mediated by oxytocin uterine-type receptors located on the cell bodies of oxytocinergic neurons. These receptors activate oxytocinergic neurons by a Ca^2+^ influx in oxytocinergic cell bodies [6,27,28,29,30], leading to the activation of NO synthase and to an increased NO production. NO, in turn, activates oxytocinergic neurons to release oxytocin in the spinal cord and extra-hypothalamic brain areas, e.g., a mechanism similar to that reported above for dopamine receptors. In fact, oxytocin injected into the PVN at a dose that induces penile erection also increases NO production in the PVN, and both these effects are prevented either by the blockade of N-type voltage-dependent Ca^2+^ channels or by NO synthase inhibitors injected into the PVN ([6,28,29] and references therein). In line with these earlier findings, a recent work shows that oxytocin is released in different brain areas, including the PVN of male mice, during copulation by means of a genetically encoded G-protein-coupled receptor-activation-based (GRAB) oxytocin sensor, with N-type and L-type calcium channels mediating axonal and somatodendritic oxytocin release, respectively [154].

A main pro-erectile role of oxytocin and its neurons is also supported by studies showing that (1) male rats selected for a low level of sexual activity with receptive female rats have significantly lower oxytocin mRNA and NO synthase mRNA contents in the PVN than those found in the PVN of male rats with a normal level of sexual activity [155,156]; (2) the blockade of central oxytocinergic receptors almost completely abolishes noncontact erections, male rat copulatory behavior and the facilitative effect of dopamine agonists on male rat sexual behavior ([6,28,29,30] and references therein); and (3) sexual interaction increases Fos, the gene product of the immediate early gene *c-fos*, in PVN oxytocinergic neurons projecting to the spinal cord that control penile erection [157].

Oxytocin induces penile erection also when injected in the hippocampus and the VTA, which contains the cell bodies of mesolimbic dopaminergic neurons ([21] and references therein) (see also Section 3.1.2.2). Both areas contain oxytocin nerve endings of neurons originating in the PVN and oxytocin receptors [158,159,160]. In the VTA, oxytocin receptors activate not only mesolimbic dopaminergic neurons but also neural pathways, which are yet to be identified and stimulate PVN oxytocinergic neurons projecting to the spinal cord, that mediate penile erection [145,148]. In the hippocampal ventral subiculum oxytocin receptors also activate mesolimbic dopaminergic neurons, increasing dopamine release in the nucleus accumbens [146,147], the effects of which are mediated by the activation of glutamic acid neurotransmission in the VTA [147,149].

Together, the above results suggest that oxytocin receptors in the PVN, VTA, hippocampus and the spinal cord make oxytocinergic receptors a target for ED treatment in men. Accordingly, in humans, plasma oxytocin levels are increased during sexual activity, mainly at ejaculation [161,162], and during the manipulation of breast and genitalia, which usually occurs during sexual intercourse [163]. However, oxytocin is unable to cross the brain–blood barrier and is easily destroyed by plasma endoproteases and brain peptidases [164,165,166,167]. This makes difficult the use of systemic oxytocin to activate its receptors in the brain and in the spinal cord to obtain central effects for not only sexual but also for the other functions in which the peptide has been involved ([21] and references therein). In fact, oxytocin is unable to induce penile erection when given systemically, although some facilitative effect on sexual behavior has been found in male rats [28]. Even when given intranasally, a route of administration that should allow oxytocin to cross the blood–brain barrier and reach the central nervous system ([168], but see [169,170]), oxytocin has been found ineffective in improving sexual behavior and its parameters in men under different experimental conditions [171,172,173]. Other administration routes must be developed to make oxytocin able to reach the central nervous system intact and in active amounts ([21] and references therein). Alternatively, non-peptide oxytocin receptor agonists able to cross the blood–brain barrier might be used instead of the peptide. In this regard, only a few non peptide compounds labelled as oxytocin receptor antagonists have been described (e.g., L-368899; [1-((7,7-Dimethyl- 2(S)-(2(S)-amino-4-(methylsulfonyl) butyramido)bicyclo [2.2.1]-heptan- 1(S)-yl)methyl) sulfonyl)-4-(2-methylphenyl) piperazine]) [173]; and cligosiban (5-[3-[3-(2-chloro- 4- fluorophenoxy)azetidin-1-yl]-5-(methoxymethyl)-1,2,4-triazol-4-yl]-2-methoxypyridine) [174,175]. The latter has been tested for use in the therapy of premature ejaculation with contrasting results ([21] and references therein).

##### 3.1.2.4. ACTH-MSH Receptors

ACTH-MSH-related peptides induce penile erection by acting in the hypothalamus (for a review, see [30,116,176]). Their pro-erectile effect was discovered in 1960, when ACTH and α-MSH were found to be able to induce penile erection and ejaculation in several laboratory animals (dogs, cats, rabbits, rats, mice and others). These sexual effects were usually seen together with the so-called “stretching-yawning syndrome”, after their central, but not peripheral, administration ([30,177] and references therein). It was then unknown that ACTH, α-MSH and ß-endorphin derive from the common precursor pro-opiomelanocortin. Since then, pro-opiomelanocortin-containing neurons were identified in the brain ([178] and references therein) and ACTH–MSH receptors have been characterized and found to be linked to AC in the adrenal gland [179]. However, it proved difficult to find specific receptors for these peptides in the central nervous system until 1992, when molecular biology studies led to the cloning of ACTH–MSH receptor genes, which encode at least five subtypes of functional high-affinity melanocortin (MC1, MC2, MC3, MC4 and MC5) receptors for ACTH–MSH peptides in several brain areas, including the hypothalamus, midbrain and brainstem, which contain mainly MC3 and MC4 receptors [180,181,182,183]. These MC receptors are coupled to AC–cAMP- or to phosphatidyl-inositol/Ca^2+^-mediated signaling pathways [182,184]. This led to the synthesis of new α-MSH analogues with high agonist and antagonist potency and selectivity at specific receptor subtypes [185,186,187] (Table 4).

One of these agonists, melanotan II, when given subcutaneously at the dose of 0.025 mg/kg to test its melanotropic activity, was found to be capable of inducing repeated penile erection episodes in human male volunteers [185] and in men with psychogenic impotence, an effect shared also by its carboxylate derivative PT-141 (bremelanotide) [187,188]. As MC3 and MC4 receptors are the only MC receptors present in the hypothalamus, it is likely that the pro-erectile effect of ACTH–MSH peptides is mediated by these MC receptor subtypes [176,189,190]. As for the neural pathways activated by MC3 and/or MC4 receptors that led to penile erection, it has been suggested that ACTH–MSH peptides also activate central oxytocinergic neurotransmission [116,190]. However, such evidence is very modest, as these peptides induce penile erection when injected not only in the PVN but also in all the periventricular area surrounding the third ventricle [176], and their pro-erectile effect is not influenced by the ablation of oxytocinergic neurons [191], or by the classical oxytocin receptor antagonists [192], nor the increase in intracavernous pressure induced by α-MSH is antagonized by a classical oxytocin receptor antagonist in contrast to that induced by oxytocin [193], although it is abolished in male rats pretreated with a NO synthase inhibitor [194]. In contrast to the above studies, the pro-erectile effect of THIQ, a non-peptide putative MC_4_ receptor agonist found to be able to induce penile erection when injected systemically in male rats, was found reduced by 20% by L-368899, a non-peptide oxytocin receptor antagonist [30,190], and MC4 Receptor knockout mice showed an increased ejaculation latency compared to control mice, which was restored by re-expression of MC4 receptors in a subset of single-minded 1 (SIM1) neurons positive for oxytocin [195]. Whatever mechanism is activated by ACTH–MSH peptides and non-peptide derivatives, their ability to induce penile erection after systemic administration in men as well makes MC3 and MC4 receptors a valuable target for ED treatment, justifying why some of these derivatives underwent clinical trials for their approval as drugs labelled for ED therapy [196,197,198,199]. However, acute priapism has been reported to occur in two recent case reports in two men using melanotan II for sunless tanning [200,201]. Since melanocortin analogues including melanotan II are illegally used for sunless tanning, these reports suggest that these compounds must be tested carefully to avoid undesired and severe collateral effects before their use in therapeutic applications [8].

## 4. Non Pharmacological Treatments of ED

Pharmacological (oral and local) ED therapies can provide help only if the main involved neural, vascular and hormonal mechanisms are still relatively intact and functioning in a satisfactory mode. However, due to aging and the increase in chronic diseases that accompany advanced age and impair erectile mechanisms, pharmacological therapies, including PDe5 inhibitors, are unable to help all men with ED. Thus, when pharmacological treatments are found scarcely efficacious or totally inefficacious in restoring the erectile response, other strategies must be used in order to attempt to overcome ED, at least if using of the old vacuum erection device or the surgical implant of a technologically advanced penile prosthesis is not an available option. This requires the identification of the main cause of the dysfunction, leading to the search of “restorative/regenerative” strategies of erectile function, which vary depending on the main cause(s) of the dysfunction. The restorative/regenerative strategies under investigation today include nonsurgical procedures, such as stem cell-based therapies and platelets-enriched plasma aimed at the restoration of cavernous endothelial cells and nerves; low-intensity extra-corporeal shock wave therapies; the latest therapy, intracavernous injection of botulinum neurotoxin A; and invasive surgical procedures at the level of the pelvic vascular (arterial and/or venous) bed, aimed at the restoration of a satisfactory in- and out-blood flow during penile erection and the implant of nerve grafts in the pelvic area (Table 5 and Table 6).

### 4.1. Stem-Cell-, Platelet-Rich-Plasma- and Gene-Transfer-Based Therapies

The pharmacological therapies reviewed above are aimed at providing symptomatic relief to ED, thus providing a temporary resolution of the problem rather than a cure aimed at resolving the cause of the dysfunction. Due to the existence of an elevated number of difficult-to-treat patients affected by ED that is often secondary to the presence of other pathologies (i.e., diabetes),lesions of pelvic and cavernous nerves as a consequence of surgical interventions at the pelvic level (i.e., partial or radical prostatectomy and radiotherapy for cancer control) or trauma of the pelvic cavernous nerves and/or the vascular system of the genital apparatus, treatment approaches of regenerative medicine developed in the last 20 years have been tested for ED. These treatments aim to find a reliable and long-lasting cure of ED with the recovery of physiological functions by reducing/eliminating the causes underlying the dysfunction rather than providing a symptomatic treatment on demand. These new therapies (at the moment, experimental only) are based on the use of stem cells, platelet-rich plasma, gene transfer and tissue engineering for the restoration of viable cavernous muscle, vascular and endothelial cells, and nerves leading to the recovery of erectile function.

The majority of studies that have used these regenerative treatments for ED have been made with stem cells of different type (e.g., mesenchymal stem cells, placental-matrix-derived stem cells, mesenchymal-stem-cell-derived exosomes, adipose-derived stem cells, bone-marrow-derived mononuclear stem cells, umbilical cord tissue or blood stem cells, and urine derived stem cells) ([202,203,204,205,206,207,208,209,210,211,212,213] and references therein). In these studies, different animals, mainly rat models of ED, have been used. These include aged rats, diabetic rats, cavernous-nerve-injured rats, and penile trauma, Peyronie’s disease and radical prostatectomy animal models. Once prepared, stem cells are transplanted in the animals by standard intracavernous injections, and the animals are evaluated to ascertain whether stem cells transplantation was successful (by measuring cell surface markers by immunochemistry and/or other methods); and whether the changes in functions (such as angiogenesis, apoptosis, smooth muscle actin) and for erectile function in vivo (measurement of intracavernous pressure) or in vitro (cavernous smooth muscle strips); and the biochemical markers of erectile function (levels of cavernous corpora cells, endothelial and neuronal NO synthase, GC, protein kinases, phosphatase, PDes, protein channels, receptors and others) had occurred. These studies have revealed that stem cell treatment has a good efficacy on ED in the tested animal models and a safe profile, but studies on the protocols and dosages of the different type of stem cells to be injected, and mechanism of action as well, are still lacking. Nonetheless, from these studies, it appears almost unanimously that stem cells induce their positive effect on ED and its altered markers by acting with a paracrine mechanism (e.g., by releasing humoral agents that allow damaged tissue components to recover, at least in part, their physiological function) rather than after being transplanted and differentiated in penile tissues. This may explain, in part, why a short lived effect is often observed in these animal models after stem cell therapy [214].

The more recent studies describe combinatory treatment approaches, with stem cells having been modified or supplemented with angiogenic and/or neurotrophic genes and proteins, such as those present in platelet-enriched plasma itself, depending on the animal model used or bioengineered with particular substances (i.e., hydrogels), with the aim to facilitate stem cell adherence in the damaged penile tissues and improve their therapeutic efficacy [207,214,215]. In these studies a positive synergic effect between stem cells and the modification added to them, usually confirming a good efficacy on ED and a safety profile [203,204,205,206,207,213]. However, due to the complications added to the stem cells preparation by the procedures required for modifying stem cells, it has yet to be determined if these modifications really increase the success of this already complex ED therapy when compared to the use of stem cells alone.

Although numerous basic studies are available in rodent models of ED, which support an improving effect of stem cell treatments on ED, very few clinical trials in men are present in the available literature. Two recent reviews on these studies identified nine trials with published results for a total of less than 100 patients included for ED treatment in Phase I and Phase II, and with follow-up periods from 6 to 62 months. End points of the studies include safety, tolerability and efficacy of stem cell therapy for ED. The majority of these studies show ED improvement due to stem cell therapy in patients, as indicated by increase in penile vascular flow, International Index of Erectile Function-15 items, and Erectile Hardness Scale scores. All studies also reported that no serious adverse effects were observed in treated patients. The main limitation of these studies was the small cohort sizes [216,217,218].

Gene transfer has also been tested for the therapy of ED. This technique allows for the insertion of a gene in a defective tissue cell in order to have it working properly and/or in a potentiated manner. Gene transfer is realized by using a vector (usually a virus or a plasmid) that enables the passage of the gene inside the cells in order to have the inserted gene working appropriately. At variance from numerous studies with stem cells, only a few studies on the use of gene transfer for ED are available. These few studies describe the gene-transfer-assisted upregulation of the “Maxi-K” potassium channel into penile smooth muscle cells to increase their relaxant properties [219,220]. “Maxi-K” potassium channel transfer to penile tissue has also been found to increase sexual behavior in atherosclerotic cynomolgus monkeys [221]. However, only data from a small number of patients with ED of different etiology are available that show the improvement of ED without adverse effects [219]. The “Maxi-K” potassium channel gene has been also transferred in mesenchymal stem cells for the treatment of diabetes-associated ED in a diabetic rat model, resulting in an improved effect on erectile function of diabetic rats [222].

In view of the very low number of clinical trials and of patients treated with this kind of therapy and in spite of the encouraging positive effects reported by the available studies, it is evident that many other studies are necessary to realize standard protocols, the dosage of cells to be injected and to identify the type of stem cell to be used with ED of different etiology. Although fascinating, the development of a therapy for ED based on strategies of this kind (complex, laborious and expensive) still appears to be difficult to realize, and far away from being realized, in a short time.

### 4.2. Low-Intensity Extracorporeal Shockwave Therapy

Low-intensity extracorporeal shock wave therapy (LI-ESWT) is a method to transfer low-intensity acoustic energy (<0.2 mJ/mm^2^) to tissues of different types, similar to the traditional Extracorporeal Shock Wave Lithotripsy used for the elimination/management of urinary stones. The first study on the use of this method with application to penile tissues for the treatment of ED appeared in 2010 [223] after the technique was tested in other soft tissues, mainly on vascular function. This study reported improving effects of low-intensity extracorporeal shock waves on cavernous hemodynamic disorders with no adverse effects. Since then, numerous studies have used this method to treat ED mainly of vascular and neurogenic origin, becoming a noninvasive therapeutic option to treat ED. The majority of these studies confirm the ameliorative, long-lasting effects of this treatment on erectile function as determined by the use of the International Index of Erectile Function—Erectile Function domain (IIEF-EF) and the Erection Hardness Score (EHS) questionnaires, when compared to sham therapy. According to these studies, at the used intensities and at the appropriate dosing, energy transfer LI-ESWT induces beneficial effects in soft tissues, such as neovascularization, tissue regeneration (including nerve regeneration) and reduction of inflammation. These effects are explained with increases in the expression of vascular endothelial growth factor, endothelial NO synthase, proliferating cell nuclear antigen, and the upregulation of chemoattractant factors and recruitment/activation of stem/progenitor cells [224,225,226,227,228]. However, against these studies, other studies showed very modest effects of LI-ESWT on ED, with its efficacy depending of the degree of severity of the dysfunction (e.g., higher the severity, lower the effect) ([228,229,230] and references therein). Due to these conflicting results and the lack of unanimously accepted protocols that specify the intensity, frequency and duration of the treatment, although appearing promising, LI-ESWT still remains an experimental approach for ED, with more clinical studies and standardization of the methodology being required for its approval and acceptance by Institutions as the Food and Drug Administration and others for its use in the therapy of ED.

### 4.3. Botulinum Neurotoxin A

The latest nonsurgical strategy for ED treatment is the intracavernous injection of botulinum neurotoxin. In fact, five years ago this neurotoxin was reported to be able to induce penile erection after intracavernous injection, given alone or in association with a PD5 inhibitor [231]. This work shows that botulinum neurotoxin A (BOTOX, 50 U), given intracavernously alone or in association with a PDe5, was able to induce penile erection in patients affected by ED and who were unresponsive to the other available therapies and selected for penile prosthesis implantation. Part of the treated patients were also found to be able to engage in sexual intercourse with the help of sildenafil, and a few of them were even able to complete it [40]. According to this study, the treatment effect lasted for more than three months with no reported collateral effects. The findings of this Phase I trial, have been confirmed and extended by other studies in patients with different botulinum neurotoxin A formulations and doses [41,231,232,233] and are corroborated by animal studies [40,234], which support the promising role of intracavernous botulinum neurotoxin A in ED treatment. The mechanism by which botulinum neurotoxin A facilitates the relaxation of cavernous smooth muscles has not been clarified by the above studies. It is likely that the neurotoxin induces its effect by blocking the release of neurotransmitters that keep cavernous smooth muscles contracted (i.e., noradrenaline, endothelin, and others) through mechanisms similar to those well characterized at the level of other neuronal synapses, mainly the cholinergic synapse in the neuromotor plaque ([235] and references therein), although other mechanisms cannot be ruled out. Although promising, at the moment treatment with botulinum neurotoxin A has to be considered experimental only and as a last resource for patients not responding to either pharmacological or surgical treatment for ED.

### 4.4. Vacuum Erection Devices

Vacuum erection devices are recalled here because they are a nonsurgical strategy for ED treatment. In fact, the idea of using a device made with a closed-end cylinder, vacuum pump, and constriction ring to obtain an erection satisfactory for sexual intercourse dates back to the end and the beginning of the 1900, when in 1917, the first vacuum erection device was introduced into clinical practice [236]. However, the first vacuum erection device that was marketed as an erectile aid approved by the U.S. Food and Drug Administration only appeared in 1968 [237]. Since then, numerous vacuum erection devices have been marketed to be used as a primary therapy for organic difficult-to treat ED, although more recently, their use in postoperative penile rehabilitation protocols, penile lengthening before penile prosthesis implantation, and as an adjunctive therapy for Peyronie’s disease, has been promoted and documented [238].

The use of vacuum erection devices in the conditions recalled above are supported by studies in animal (rat) models of ED [239,240] and clinical studies in human penile (cavernous and vascular) tissues aimed at characterizing the main effects of the use of the device on physiological markers of erectile function. These studies have ascertained that the vacuum chamber creates an erection by generating negative pressure within the penis, increasing blood flow, and distending the corporeal sinusoids [240,241]. The application of a constriction ring at the basis of the penis maintains the erection allowing penetration [242]. Oxygen saturation studies demonstrate that blood supply to the penis induced by vacuum device is mixed arterial and venous in origin [239,243]. These studies have also revealed that penile oxygenation in the presence of the constriction ring decreases with time reaching ischemic values 30 min after the application of the ring [243], in line with the fact that vacuum device-induced erection should only be maintained for periods of time shorter than 30 min to avoid ischemic damage and associated complications. Studies have also produced molecular data showing that the use of vacuum devices reduces the levels of hypoxia-inducible factor-1a, transforming growth factor-b_1_, collagen, and apoptosis, which occur concomitantly to an increase in the levels of endothelial NO synthase and α-smooth muscle actin [241].

Altogether, the above findings suggest that the use of vacuum devices increases penile blood flow, improves tissue oxygenation, preserves or helps to recover tissues relevant for maintaining erectile function, and suppresses apoptosis and fibrosis. In view of the modest collateral effects of vascular devices, e.g., transient penile numbness, pain, and ecchymoses (at the site of the constriction ring), lower penile temperature and sensation and of the few contraindications to the use of the device (e.g., bleeding disorders and priapism), several researchers have suggested a role for this device in post-surgical penile rehabilitation in order to preserve erectile function, maintain tissue architecture, and decrease venous occlusive dysfunction and ED [244].

Several studies have reported a high success rate of vacuum devices in achieving erection in patients and satisfactory sexual intercourse with their partner in ED of various etiology (psychogenic, spinal cord injury, diabetes mellitus, prostatectomy and other organic causes) [244,245,246,247,248,249,250]. This picture has changed after the discovery of oral PDe5 inhibitors and their introduction in the therapy of ED. Since then, in fact, in spite of the high success in allowing erection and sexual intercourse, the use these devices has been replaced by the oral therapy due to its easiness, allowing for higher compliance by patients, at least when efficacious, leaving the use of vacuum devices, alone or in combination with the oral and intracavernous therapies, to those cases in which the latter therapies are found scarcely effective or totally ineffective on ED or to their use in postoperative penile rehabilitation protocols, penile lengthening before penile prosthesis implantation, and as an adjunctive therapy for Peyronie’s disease, as recalled above [238]. However, this has not blocked preclinical basic research in animal models aimed at improving the utilization parameters of this device [251] to be used for difficult-to-treat ED, as it occurs in organic, vasculogenic, trauma-mediated, postoperative prostatectomy and radiotherapy for controlling cancer of the urogenital apparatus.

### 4.5. Surgical Treatment of ED

The occurrence of a penile erection adequate for vaginal intromission and sexual intercourse requires sufficient arterial blood supply to, and a venous occlusion mechanism from, the cavernous and spongiosum corpora of the penis. Alterations in blood supply (arterial insufficiency) and/or venous occlusion mechanism (blood flows out from penile tissues in spite of an adequate arterial inflow to get penile erection) are among the main causes of ED and can occur either in the presence of vascular risk factors often associated with ED, such as hypertension, diabetes, cardiovascular disease, atherosclerosis and dyslipidemia, but also as a consequence of physical traumas and surgery intervention (i.e., prostatectomy), which may damage not only pelvic and perineal blood vessels (vasculogenic ED) but also penile nerves (neurogenic ED) ([238,252] and references therein). When the cause is ascertained by means of penile duplex Doppler ultrasonography and/or dynamic infusion cavernosometry and cavernosography, ED secondary to arterial insufficiency may be treatable through surgical procedures. These aim at restoring penile vascularization by creating an arterio-arterial shunt between the inferior epigastric artery to the dorsal penile artery and/or cavernosal artery, an arterio-venous shunt between the inferior epigastric artery and the deep dorsal penile vein, or a venous arterialization through anastomosing the inferior epigastric artery to the deep dorsal vein (see [238,252]). When ED is due to venous occlusion alterations, venous ligation or embolization have been described to interrupt at least one of the branches of penile and pelvic veins, thereby preventing retrograde drainage or glanular venous congestion, respectively, at least partially [253,254,255]. Positive results have been reported with these procedures in a few number of selected patients with an age not higher than 55 years and those who did not present other vascular risk factors [238,252]. The scarce use of this kind of intervention is due mainly to the difficulties of the surgical procedures, which require expert surgeons in microvascular surgery since, even when the main cause of ED is well known, it is often difficult to identify the surgical procedure that would produce the better effect on the specific ED to be treated. Recently, penile microarterial bypass surgery with or without a drug-eluting stent implant surgery, which is similar to the very efficacious procedures used worldwide for coronary arteries, have been added to the above procedures for arterial insufficiency in ED [256,257,258,259,260]. Despite these surgical procedures possible being thought of as the only treatment capable of restoring arterial blood inflow and a close-to-normal erectile function without long-term use of vasoactive medications or penile prosthesis placement, they are still considered experimental due to the need of elevated vascular microsurgery expertise, the lack of standardization in patient selection, hemodynamic evaluation, surgical technique, and limited long-term outcome data using validated instruments [261]. Nonetheless, the available studies with microarterial bypass surgery with or without a stent suggest that these procedures may be extremely effective as a long-lasting treatment of focal vasculogenic ED only in young men (age lower than 55 years) without other vascular risk factors [238,252,260].

Procedures aimed at reconstructing damaged nerves at the pelvic and cavernous level have been also described. These procedures are based on cavernous nerve graft reconstruction, which represents a dramatic challenge for every surgeon. Sural nerve grafts and/or neurorrhaphy have been used to reinstate lesioned nerve function at the cavernous level after pelvic trauma and after radical prostatectomy [262,263,264,265,266], which remains one of the major causes of ED in spite of the use of robot-assisted nerve sparing surgery compared to conventional surgical laparoscopy/open surgery methods. In fact, irrespective of the surgical technique used, prostatectomy causes disruption of normal pelvic anatomy and alters both the nerve and vascular supply to the penis [267,268]. Additionally, in this case, few studies are available, and the number of patients is very low. Although showing positive results on ED, this type of surgical procedure also needs to be further investigated and requires expert surgeons in microsurgery and cavernous nerve graft reconstruction before they can be used routinely in clinical practice. In summary, the available studies on surgical interventions suggest that these procedures may represent a way to provide long-term therapy for ED when the cause of the dysfunction is vasculogenic and well known (focal damage/obstruction) in young patients who do not present other vascular risk factors secondary to pathologies, such as hypertension, diabetes, atherosclerosis, and others. However, their application is complicated by the paucity of criteria for patients selection, by the intrinsic difficulties in the procedures themselves that need high expertise in vascular microsurgery and nerve graft reconstruction, and by the fact that, even when the cause of ED is well known, it is difficult to identify the procedure which would produce the best results in terms of erectile function recovery. Thus, more work is required to make these complex procedures accessible to patients affected by vasculogenic and/or neural damage ED. In this regard, a recent study has described the intraoperative implant of a two-dimensional flexible electrode array that covers the entire plexus area, ensuring that at least one of the electrodes will be in optimal contact with the cavernous nerve, without the need of intraoperative identification, in 24 patients enrolled for radical prostatectomy. Electrical stimulation was found to be able to induce penile erection as assessed by visual change of penile tumescence and by penile plethysmography in 75% of patients, with the quality of the erectile response correlated with the values of the International Index of Erectile Function-5 score test, with lower scores associated with reduced erectile response [269]. Although this study confirms that electrical stimulation of the cavernous nerve induces penile erection, these results have been obtained in an intraoperative condition, and it is unknown if this kind of intervention also will work in early prostatectomized patients.

### 4.6. Penile Prosthesis

The surgical implantation of a penile prosthesis is not a new surgical intervention for ED treatment, as it has been used to treat ED regardless of its etiology since 1973 [270]. It is usually used on patients for whom the other available therapies (including PDe5) are found inefficacious. Several prosthetic devices are available that have been continuously technically implemented and made more reliable, safe and long lasting. The latest three-piece inflatable penile prostheses have the advantage of simulating the natural process of erection, as they can be activated to make the penis erect and deactivated to make the penis flaccid when not in use. These prostheses are also reported to have one of the highest patient satisfaction rates among all medically implanted devices (from 85 to 90%) and the lowest mechanical revision rate [268,271,272,273,274,275], with more than 90% of implanted patients being able to achieve normal sexual activity with their partners [276]. Several studies have also shown that penile prosthesis implantation is particularly suitable for ED patients affected by Peyronie’s disease [277] and for patients who underwent radical prostatectomy [273]. However, against the above advantages, penile prosthesis implantation is highly expensive, traumatic (cavernous tissue is irreversibly damaged by the implantation procedure), and may also present severe, although rare, complications, such as prosthesis infection, pump migration and automatic inflation, which can require a second surgery and other interventions. These complication may be reduced by appropriate patient selection, strict adherence to antimicrobial prophylaxis and safe surgical practice, and preoperative informative counseling is essential for the patients in order to reach postimplantation satisfaction with their partners [274,275,278,279,280,281].

## 5. Final Remarks

The discovery of orally active PDe5 inhibitors 30 years ago has represented the main worldwide breakthrough for the therapy of ED. Their success across the world has made these drugs the main therapy of ED. This is valid even today, when it is known that not all men with ED obtain a satisfactory improvement of their dysfunction with these compounds, and that in some cases, PDe5 inhibitors are inefficacious. This led to the search for new therapeutic strategies for these forms of difficult-to-treat and/or intractable ED. PDe5 inhibitors’ failures usually occur when vascular and neural supply to the penile cavernous smooth muscles is impaired, i.e., in pathological conditions (hypertension, diabetes, atherosclerosis, dyslipidemia) or after prostatectomy, pelvic surgery, pelvic and/or penile trauma. These conditions usually induce dramatic changes in the morphology, physiology and neurophysiology of all penile tissues. Among the changes that may be responsible for the failures of PDe5 inhibitors in ED treatment are those that lead to a low content of NO in cavernous tissue (i.e., decrease in NO synthase content in the endothelial cells overlying smooth muscle cells and nitrergic synapses, low levels of the NO synthase substrate L-arginine, etc.). Several pharmacological strategies have been, and are still being, explored to overcome this condition. The first is the administration of NO donors clinically used for inducing vasodilation in several clinical conditions [282,283,284]. Since these drugs exert their effect on the entire vascular system, NO donors coupled with a PDe5 inhibitor have been synthesized to have NO released selectively in cavernous tissues. Some of these compounds have been found more effective on cavernous smooth muscle than the PDe5 inhibitor alone in experiments in vitro [36,53,54,55,56]. However, to our knowledge, no further progress has been reported for this kind of drugs. A new strategy, recently tested to have NO donors releasing NO only in the penile tissues, is the use of light-controllable NO donors, compounds that release NO when activated by light at a given wavelength. A few of these new NO donors are able to induce cavernous smooth muscle relaxation in vitro and penile erection in anesthetized male rats when injected intracavernously [57,58,59,60]. However, it has still to be found how to maintain penile erection once intromission has occurred and light cannot activate the NO release anymore [59,60].

NO levels may also be increased by reducing arginase 1 and 2 activity in penile tissues. As these enzymes convert L-arginine to urea and L-ornithine and compete with NO synthases for L-arginine [76], they are a target for the therapy of vascular ED, as found in diabetes and atherosclerosis [76,80,81]. However, although several arginase inhibitors are available [38,87], doses and administration routes have yet to be identified to test these compounds in vascular pathologies including ED, as has already been done with other polyphenols in cardiovascular diabetology [88,89,90].

The direct stimulation of GC to increase cGMP levels independently of NO levels has also been investigated to induce cavernous smooth muscle relaxation using soluble GC stimulators/activators, which bind to a site of the GC molecule independently of NO [62,63,64]. Although a few of these compounds induce relaxation of cavernous smooth muscles and induce penile erection in vivo by increasing cGMP levels [36], when given systemically, these drugs also caused a marked decrease in blood pressure and inhibited platelet aggregation [75]. It seems unlikely then that these types of drugs, when given systemically, might be used for the therapy of ED unless pro-erectile GC stimulators/activators unable to decrease blood pressure are discovered.

RhoA/Rho kinase system inhibition has been also evaluated to induce cavernous smooth muscle relaxation independently of NO with RhoA/Rho kinase inhibitors that induce penile erection in vivo in male rats ([91,92] and references therein), and in diabetic rabbits and rats in a NO-independent manner [99,100]. However, despite the large body of experimental evidence supporting the role of RhoA/Rho kinase inhibitors in improving erectile function in pathological conditions [91], no advancements in the therapy of ED have been obtained with these drugs so far. This may be due to the marked decrease in systemic blood pressure that occurs when these drugs are given systemically because of the presence of the RhoA-Rho kinase system in all vascular beds.

From what has been discussed above, it is evident that pharmacological strategies aimed at overcoming PDe5 inhibitors’ therapy and their failures have produced very little progress for the therapy of ED so far. Although it may be argued that the majority of the tested strategies have produced drugs aimed at increasing NO–GC–cGMP pathway activity with potent systemic effects, mainly on systemic blood pressure, which are incompatible with the ED therapy, it is important to keep in mind that many changes occur in penile tissues in addition to those influencing the NO–GC–cGMP pathway in pathological conditions or after prostatectomy, pelvic surgery, pelvic and/or penile trauma, which may lead to ED. Unexpectedly, at least until now, modest clinical progress that is useful for ED therapy has also been obtained with the restorative/regenerative strategies based on the use of stem cells, platelet-enriched plasma or gene transfer developed to produce a long-lasting recovery of penile cavernous smooth muscle, vascular and/or neural functions required for penile erection. In fact, in spite of a large amount of experimental work in animal, mainly rat models, of ED (from aged and diabetic rats to radical prostatectomy rat models) with the intracavernous injection of stem cells obtained from numerous tissues, these studies have only shown that stem cells improve ED by acting with a paracrine mechanism (e.g., by releasing humoral agents that allow damaged tissue components to recover, at least in part, their physiological function) rather than after being transplanted and differentiated in penile tissues. Not much better results have been obtained with stem cells modified or supplemented with angiogenic and/or neurotrophic genes, proteins such as those present in platelet-enriched plasma and with the intracavernous injection of platelet-enriched plasma itself, or stem cells bioengineered with particular substances (i.e., hydrogels), aimed to facilitate stem cell adherence and implant in damaged penile tissues to improve their therapeutic efficacy [206,213,214207,214,215). So far, data from only about one hundred patients who underwent stem cell therapy are available. These clinical data confirm a promising effect of this form of ED therapy, but there are still very little, so stem cell therapy for ED still has to be considered highly experimental. Many other studies are required to realize standard protocols and the dosage of cells to be injected and to identify the type of stem cell to be used with ED of different etiology. Although fascinating, the development of a therapy for ED based on strategies of this kind (complex, laborious and expensive) still appears to be difficult, and far away from being realized and used routinely for patients in a short time.

Scarce progress in the therapy of ED has also been obtained with the LI-ESW therapy since its first application for ED in 2010, reporting the improving effect of this method on cavernous hemodynamic disorders with no adverse effects [223]. Although several studies suggest that the improving effects of LI-ES waves occur with increases in the expression of factors involved in tissue regeneration [224,225,226,227,228], other studies show very modest effects of this therapy on ED and revealed that its efficacy was strictly related to the degree of severity of the dysfunction (e.g., higher the severity, lower the effect of LI-ESW treatment) ([229,230,285] and references therein).

As for the other available methods to restore penile erection in difficult to treat ED, some progress has been obtained in surgical procedures aimed at restoring arterial blood inflow to the penis in the presence of arterial obstruction, mainly at the level of the inferior epigastric artery, with the use of stents (as is routinely done for coronary arteries) and reducing a dysfunctional venous occlusion mechanism by venous ligation or embolization. However, the most recent studies in this field have also revealed that revascularization of the penis restores erectile function, resolving ED only when obstructions are focal and patients are young, age < 55 years, and do not present other vascular risks. Thus, when diffuse vascular damage has occurred, these complex microsurgical interventions are often short-lasting and inefficacious in restoring erectile function [257,260].

The modest progress provided by the strategies reviewed above has left intracavernous therapy of ED with the “old” drugs (phentolamine, prostaglandin E1, alprostadil and papaverine and their combinations) as the second line treatment for ED when PDe5 inhibitors are inefficacious and in other conditions that cause intractable ED. The only relevant modification of this therapy is the intraurethral administration that has been realized for these drugs, which eliminates the needle injection in the penis and associated discomfort (but see [284]). Thus, even today, vacuum erection devices (introduced in 1968) and the implant of a modern technically advanced inflatable three-pieces penile prosthesis (introduced in 1990) remain the last available solution to overcome intractable ED. When feasible, these “old” strategies are reported to have a high success rate (even 90%), allowing sexual satisfactory intercourses. Penile prosthesis has the certain advantage of simulating the natural erection process against the mechanical manual procedures required for the use of vacuum devices (which cause numerous dropouts in the users), but is an irreversible and much more expensive treatment and requires more cautions for its use compared to vacuum devices. whether this picture is going to change in the near future is unknown. Starting just five years ago, a few studies have shown that botulinum neurotoxin A injected intracavernously was able to induce penile erection in patients with intractable ED and made them able to engage in sexual intercourse, and even to complete it, with the help of a PDe5 inhibitor. The effect was long-lasting (three–six months) with no collateral effect [40,41,231,232,233,234]. These studies suggest that cavernous smooth muscles may be the target for this effect of botulinum neurotoxin A. Although the results of these studies have to be considered as only preliminary, and it is unknown if they will lead to the clinical use of intracavernous botulinum neurotoxin A for otherwise intractable ED, it would be really surprising to discover that ED may be treated as a cosmetic problem.

In conclusion, orally active PDe5 inhibitors are still the mainly used therapy of ED worldwide today. Their success has also driven researchers working in this field to focus on investigating the role of the NO–GC–cGMP pathway and other erectile processes at the penile level, thus reducing the interest for other mechanisms that may also play a role in ED. Among these are central mechanisms including those for which there is a large body of experimental evidence to support the role in ED treatment, such as dopamine, oxytocin and ACTH-MSH peptides and their receptors. As for dopamine receptors, this has occurred even if preclinical data show that dopamine D_4_ receptor agonists devoid of emetic properties induce penile erection and facilitate sexual behavior in male rats with a potency similar to that of apomorphine, which had scarce success in ED treatment due to its emetic properties (see [8,23]). In fact, these compounds represent an alternative to apomorphine to be tested in clinical studies for ED. Oxytocin also merits to be considered for ED treatment in view of a large amount of preclinical data showing its potency in inducing penile erection in rodents and monkeys, even if it has been reported inefficacious in facilitating erectile function in men. This may be due to the inability of oxytocin to cross the blood–brain barrier in amounts sufficient to stimulate its receptors in the brain. Identifying routes of administration that allow oxytocin to reach the central nervous system intact or oxytocin analogues (peptidic or not peptidic) able to cross the blood–brain barrier that act as selective oxytocin receptor agonists may resolve this problem ([21] and references therein). As to ACTH-MSH peptides, several potent analogues that induce penile erection in men are available, but further clinical trials are necessary with these and other analogues to ascertain the absence of severe collateral effects (e.g., priapism) [200,201].

## Figures and Tables

**Table 1:** Pharmacology of ED: local strategies.

Mechanism Involved	Molecular Targets	Classes of Drugs	Clinically Relevant Drugs
Facilitation of cavernous smooth muscle relaxation	NO–cGMP signaling pathway, neurotransmitter/ neuropeptide/autacoid receptors, adenylate cyclase/cAMP signaling pathway, Ca^2+^ channels, K+ channels	PDe 5 inhibitors, NO donors, guanylate cyclase activators, Pde-resistant cGMP analogues, NO-independent GC stimulators and activators, arginase inhibitors, PDe3/4 inhibitors, contracting prostaglandins, vasoactive intestinal peptide (VIP).	Sildenafil Vardenafil Tadalafil Avenafil Prostaglandin E1 Alprostadil Papaverine
Inhibition of cavernous smooth muscle contraction	adrenergic α_1_-receptors, endothelin receptors, RhoA/Rho kinase system	adrenergic α_1_-antagonists, endothelin receptor antagonists, RhoA/Rho Kinase inhibitors, botulinum neurotoxin A	Phentolamine Phenoxybenzamine Moxisylite Botulinum neurotoxin A

Details are in references [4,6,7,8,9,10,11,12,31,32,33,34,35,36,37,38,39,40,41,42].

**Table 2:** Pharmacology of ED: central strategies.

Mechanism Involved	Molecular Targets	Classes of Drugs	Clinically Relevant Drugs
Increased activity of neurotransmitters/neuropeptides/autacoids that facilitate erectile function	Neurotransmitter/ neuropeptide/autacoid receptors, second messengers, etc.	Dopamine agonists, excitatory amino acid agonists, oxytocin agonists, nitric oxide donors, melanocortin receptor agonists, hexarelin analogues, VGF analogues	Apomorphine, melanotan II, PT-141
Decreased activity of neurotransmitters/neuropeptides/autacoids that inhibit erectile function	Neurotransmitter /neuropeptide/ autacoid receptors, second messengers, etc.	α_2_-antagonists, opioid antagonists, gamma-aminobutyric acid (GABA) antagonists, cannabinoid CB_1_ receptor antagonists, serotonin antagonists	Yohimbine, trazodone

VGF peptides derive from VGF, a protein produced by the *vgf* gene originally identified because its mRNA was selectively induced by Nerve Growth Factor in PC12 cells. Details are in references [6,7,8,11,12,13,14,15,16,17,18,19,20,21,22,23,24,25,26,27,28,29,30,113,114,115,116,117,118].

**Table 3:** Classification of dopamine receptors.

Dopamine Receptor Family	Receptor Subtypes	Transduction Mechanisms	Effect on Penile Erection
D1	D_1_	Increased activity of AC–cAMP signalling pathway, increased PIP_2_ turnover, increased Ca^2+^ mobilitation	None
	D_5_		
D2	D_2s_–D_2l_	Decreased activity of AC–cAMP signalling pathway, increased K+ channel activation, increased voltage-gated Ca^2+^ channel activation	Facilitatory
	D_3_		None
	D_4_		Facilitatory

Details are found in references [13,22,25,29,120,121,123,125,126,127,128,129,130,131,132,133,134,135,136,137,138,139,140,141,142,143].

**Table 4:** MC receptor agonists and antagonists used to prove a role of these receptors in the control of erectile function.

α-MSH	Ac-Ser-Tyr-Ser-Met-Glu-His-Phe-Arg-Trp-Gly-Lys-Pro-ValNH_2_
MT-II	Ac-Nle^4^-cyclo[Asp^5^-His^6^-D-Phe^7^-Arg^8^-Trp^9^-LysNH_2_^10^]α-MSH(4–10)
PT-141	Ac-Nle^4^-cyclo[Asp^5^-His^6^-D-Phe^7^-Arg^8^-Trp^9^-LysOH^10^]α-MSH(4–10)
SHU-9119	Ac-Nle^4^-cyclo[Asp^5^, His^6^, D-Nal(2′)^7^, Arg^8^-Trp^9^-LysNH_2_^10^]α-MSH(4–10)
HS024	(cyclo[AcCys^3^-Nle^4^-Arg^5^-His^6^-D-Nal^7^-Arg^8^-Trp^9^-Lys^10^-CysNH_2_^11^]α-MSH(3-11)
THIQ	(N-[(3R)-1,2,3,4-tetrahydroisoquinolinium-3-ylcarbonyl]-(1R)-1-(4-chlorobenzyl)-2-
	[4-cyclohexyl-4-(1H-1,2,4-triazol-1ylmethyl)piperidin-1-yl]-2-oxoethylamine

MT-II (melanotan II) and PT-141 are nonselective MC receptor agonists, while SHU-9119 and HS024 are MC3/MC4 receptor antagonists. THIQ is a nonpeptide MC4 receptor agonist. For details, see references [185,186,187,188,189].

**Table 5:** Nonpharmacological strategies for the therapy of ED: nonsurgical strategies.

Strategy	Mechanism of Action	Preclinical Evidence	Clinical Evidence
Intracavernous, normal or modified stem cells obtained from numerous tissues	Erectile function is recovered by restoring the activity of endothelial and smooth muscle cells, and nerves in penile cavernous and vascular tissues	Yes, in rodent models of ED, mainly diabetes, vascular, nerve injuries and aging models	Yes, in patients with ED of different etiology, mainly diabetes and prostatectomy
Platelet-enriched plasma	Erectile function is recovered by restoring the activity of endothelial and smooth muscle cells, and nerves in penile cavernous and vascular tissues	Yes, in rodent models of ED, mainly diabetes, vascular, nerve injuries and aging models	Yes, in patients with ED of different etiology, mainly diabetes and prostatectomy
Gene transfer	Erectile function is potentiated by inserting genes that facilitate relaxation in cavernous smooth muscle tissues	Yes, in rodent models of aging and in a monkey model of atherosclerotic ED	Yes, but only preliminary clinical data (Phase 1) are available in patients with ED of different etiology
Intracavernous botulinum neurotoxin A	Erection is obtained by a relaxation of cavernous smooth muscle by a still unknown mechanism, possibly mediated by the block of the release of mediators that keep cavernous smooth muscle contracted	Yes, in rodent models of ED, mainly hypertension model	Yes, in patients with ED of different etiology, mainly vascular, selected for penile prosthesis implant

Details are found in the Section 4.1 and Section 4.3.

**Table 6:** Nonpharmacological strategies for the therapy of ED: surgical strategies.

Strategy	Mechanism of Action	Preclinical Evidence	Clinical Evidence
Vascular surgery with or without stent	Revascularitation by eliminating focal arterial blocks mainly in the inferior epigastric artery	Yes, in rodent models of ED, mainly vascular	Yes, in patients with vascular ED caused focal arterial damage, mainly of the inferior epigastric arthery
Venous ligation or embolization	Blockage of venous branches to reduce venous outflow from the penis during erection	Yes, in rodents models of ED, mainly aging	Yes, in patients with cavernous venous occlusive dysfunction
Neuronal gafts	Reconstruction of pelvic nerves and/or reunion of damaged/truncated pelvic nerve axons to the penis	Yes, in rodent models of ED, mainly penile nerve trauma and/or crash	Yes, in patients with pelvic nerve damage, mainly after pelvic/penile trauma and radical prostatectomy

Details are found in the Section 4.5.

## Data Availability

Not applicable.
